# Review on Hydrophobic Thin Films Prepared Using Magnetron Sputtering Deposition

**DOI:** 10.3390/ma16103764

**Published:** 2023-05-16

**Authors:** Yuxin Ju, Ling Ai, Xiaopeng Qi, Jia Li, Weijie Song

**Affiliations:** 1 Faculty of Materials Metallurgy and Chemistry, Jiangxi University of Science and Technology, Ganzhou 341000, China; juyuxin@nimte.ac.cn (Y.J.); qxpai@163.com (X.Q.); 2 Ningbo Institute of Materials Technology and Engineering, Chinese Academy of Sciences, Ningbo 315201, China; lijia@nimte.ac.cn; 3 Center of Materials Science and Optoelectronics Engineering, University of Chinese Academy of Sciences, Beijing 100049, China; 4 Research Center for Sensing Materials and Devices, Zhejiang Lab, Hangzhou 311121, China

**Keywords:** hydrophobic, wettability, magnetron sputtering, surfaces

## Abstract

Hydrophobic thin films have gained significant attention due to their broad applications in self-cleaning, anti-corrosion, anti-icing, medicine, oil–water separation, and other fields. The target hydrophobic materials can be deposited onto various surfaces thanks to the scalable and highly reproducible nature of magnetron sputtering, which is comprehensively overviewed in this review. While alternative preparation methods have been extensively analyzed, a systematic understanding of hydrophobic thin films fabricated using magnetron sputtering deposition is still absent. After outlining the fundamental mechanism of hydrophobicity, this review briefly summarizes three types of sputtering-deposited thin films that originate from oxides, polytetrafluoroethylene (PTFE), and diamond-like carbon (DLC), respectively, primarily focusing on the recent advances in their preparation, characteristics, and applications. Finally, the future applications, current challenges, and development of hydrophobic thin films are discussed, and a brief perspective on future research directions is provided.

## 1. Introduction

Hydrophobic thin films have attracted significant attention in both basic research and practical applications due to their unique properties [1,2,3,4,5,6,7,8,9,10]. Over the past few decades, these thin films have undergone extensive study, and numerous efforts have been made to broaden their application fields to include areas such as oil hydrophobicity [11,12], hydrophobic anti-icing [13], and hydrophobic anti-corrosion [14,15]. Additionally, fresh studies that explore approximate theories and fabrication techniques for hydrophobic thin films have emerged [16,17,18,19].

Hydrophobic surfaces have great potential in a number of industries and biomedical fields, among others. The protection of electrical wires and antennas from snowfall, autos with self-cleaning windows, ships with anti-corrosion coatings, metal refining, building glasses with dust-free coatings, the separation of oil and water, and textiles resistant to stains are a few examples that come to mind.

Building a micro–nano rough structure on hydrophobic substrates or chemically altering a hierarchically structured surface with a low-surface-energy material are the two main methods for creating hydrophobic thin films. Many preparation methods have been reported in the literature, including phase separation [20], plasma methods [21,22], chemical vapor deposition (CVD) [23,24], physical vapor deposition (PVD) [25], sol-gel processing [26,27], and others. However, these techniques differ in terms of their efficiency, cost, simplicity of use, and requirements for specialized reagents. Some techniques are also restricted to basic laboratory experiments, and much work still needs to be performed to prepare hydrophobic films on a commercial scale. Therefore, researchers are working to make these films easier to prepare, cheaper, more durable, and more functional.

PVD is a frequently used technique for the deposition of thin films on a substrate. PVD involves the transfer of material from a source, typically in the form of a solid or liquid, to a substrate under vacuum conditions. There are several methods of PVD deposition, including sputtering [28,29], evaporation [30,31], and pulsed laser deposition (PLD).

Magnetron sputtering is a widely used PVD method for producing hydrophobic thin films. While the creation of small-area films using magnetron sputtering has been well established, the fabrication of large-area films poses unique challenges. Achieving uniformity and precise control of deposition parameters over a large area is inherently more difficult due to factors such as edge effects, variations in gas flow, and substrate curvature. In the context of magnetron sputtering, the feasibility of preparing large-area films arises from several distinctive features of the technique [32,33,34,35]. Moreover, the precise control of deposition parameters, such as gas pressure, target-to-substrate distance, and power density, plays a crucial role in achieving uniformity and high-quality films over large areas. So, this method stands out for its feasibility of preparing large-area films and is widely used in the industry.

Some other exceptional qualities, such as capacity for mass production, environmental friendliness, low cost, and powerful adhesion between film and substrate, also draw much attention. A high voltage is applied across the target material, creating a high-energy plasma that causes atoms or ions to be ejected from the surface of the target. To achieve uniform film deposition, these particles are then deposited onto the rotating substrate in a high-vacuum deposition chamber. However, a thorough analysis of magnetron-sputtered hydrophobic thin films is still lacking.

Hydrophobic films may be affected by environmental factors (such as ultraviolet rays, high temperatures, humidity, etc.) and physical or chemical effects (such as friction, corrosion, etc.) during long-term use, resulting in a decrease in or failure of their hydrophobic ability. Hydrophobic films have a number of significant characteristics that make them suitable for a variety of real-world uses. First, hydrophobic durability describes the thin film’s capacity to retain its hydrophobic qualities over time, even after exposure to environmental factors. In many applications, particularly those where water or moisture can cause damage or impair performance, hydrophobic durability is crucial. For instance, hydrophobic thin films can extend the lifespan and increase the reliability of sensitive electronics components by shielding them from water damage. Second, the ability to withstand exposure to harsh environments and to a variety of chemicals, including acids, bases, and solvents, is the second requirement for chemical stability in hydrophobic films. Additionally, they ought to be strong enough to withstand everyday wear and tear, as well as exposure to harsh temperatures and UV rays. Third, the hydrophobic film needs to be transparent in some applications, such as optics or electronics. Finally, biocompatibility, abrasion resistance, etc., are also significant in some other applications.

Therefore, in this review, we provide an overview of recent progress in magnetron sputtering films for hydrophobic applications. We specifically provide a detailed description of three different types of sputtering-deposited thin films, including oxides, PTFE, and DLC. We include information on their preparation, properties, and applications. Finally, we provide a conclusion and address future perspectives for this emerging field.

## 2. Principle of Hydrophobicity

### 2.1. Nature Inspiration

**Figure 1:**
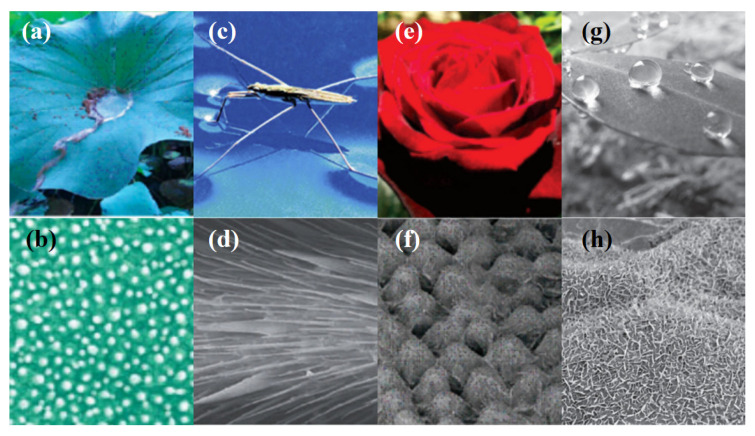
Natural hydrophobic surfaces. (**a**) Water beads formed on a lotus leaf shows the “Lotus Effect”. Reprinted with permission from ref. [7]. Copyright 2015 American Chemical Society. (**b**) Scanning electron microscope (SEM) image of the lower surface of the lotus leaf. Reprinted with permission from ref. [38]. Copyright 2002 Wiley. (**c**) Water strider legs show superhydrophobicity. Reproduced with permission from ref. [39]. Copyright 2006 Wiley. (**d**) SEM images of a water strider leg showing numerous oriented spindly microsetae. Reprinted with permission from ref. [40]. Copyright 2004 Nature Pub-lishing Group. (**e**,**f**) SEM images of the surface of a red rose petal, showing a periodic array of micropapillae and nanofolds on each papillae top. Reproduced with permission from ref. [41]. Copyright 2008 American Chemical Society. (**g**,**h**) Water droplets with spherical shape pinned on the irregular surface of peanut leaves and SEM image shows the top of nanoslices are covered with nanostructured papillae. Reproduced with per-mission from ref. [42]. Copyright 2013 Wiley.

Numerous distinctive natural surfaces, such as lotus leaves, butterfly wings, cicada wings, and rose petals, offer fresh concepts for human designers of hydrophobic thin films. The lotus leaf primarily displays excellent superhydrophobicity; when it rains, water beads form on the leaves. The water beads roll away from the leaves as long as they are slightly tilted. The “Lotus Effect,” also known as the self-cleaning effect, was first discovered in the 1970s by a group of German botanical classification scientists led by W. Bartroot. Figure 1 shows some natural hydrophobic surfaces for a better understanding. Two popular techniques for producing the “Lotus Effect” on surfaces are nanoimprinting and anodic aluminum oxide techniques. With the help of pressure and heat, a mold having nano-scaled patterns is replicated onto a polymer substrate using the nanoimprinting technique. With this method, a surface topography that resembles the roughness of a lotus leaf is produced. The fabrication of the nano mold can be performed in a number of ways, but the most popular way to create a superhydrophobic surface is to copy a natural leaf or use deep reactive-ion etching on a silicon or silicon oxide substrate [36]. On the other hand, the anodic aluminum oxidation technique is a method that uses anodized templates as molds to grow a highly ordered nanotube structure on an aluminum surface. The surface of the resulting structure is hydrophobic due to its surface roughness [37]. The “Lotus Effect” is a remarkable phenomenon that displays superhydrophobicity. This effect has inspired researchers to develop hydrophobic thin films that exhibit self-cleaning properties [38,39,40,41,42].

**Figure 2:**
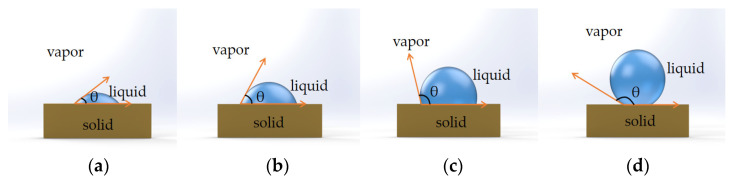
Wetting behavior of a liquid droplet on solid substrates with different contact angles. (**a**) superhydrophilic: θ < 5° in 0.5 s. (**b**) Hydrophilic: θ < 90°. (**c**) Hydrophobic: θ = 90°~150°. (**d**) Superhydrophobic: θ = 150~180°.

As a result, when creating a superhydrophobic thin film, we typically create a micro–nano rough structure and lower the surface energy of the substance, because it has been discovered that a nano multilayer structure and low-surface-energy wax together form surface superhydrophobic properties. The main reason why water drops on surfaces is due to high-energy molecules on the surface that have a strong affinity for the liquid, as illustrated in Figure 2, depicting different wetting behaviors with different contact angles. To fully comprehend how liquids adhere to solid surfaces, the contact angle must be accurately measured. The sessile drop method, the Wilhelmy plate method, the pendant drop method, and axisymmetric drop shape analysis (ADSA) are some of the techniques that have been developed to measure the contact angle. In the sessile drop method, an optical instrument, such as a goniometer, is used to measure the contact angle between a small droplet of the liquid and the solid surface. By measuring the angle between the liquid droplet’s tangent line and the solid surface at the three-phase contact point (where liquid, solid, and air meet), the contact angle can be determined. In the Wilhelmy plate method, a solid plate or rod is submerged in the liquid, and the force necessary to separate the liquid from the surface is measured. The contact angle is determined using the pendant drop method, which suspends a droplet from a needle or capillary tube. The angle between the droplet’s tangent line and the needle or capillary tube is then calculated. The shape of a suspended droplet is lastly captured with ADSA using a high-resolution camera, which is then examined using mathematical models to determine the contact angle. Using a contact angle analyzer, the water contact angles of the untreated and treated substrates can be measured to determine whether the treated substrate is hydrophilic or hydrophobic [43].

Based on the contact angle, surface wettability is also divided into four categories: superhydrophilic (completely wetting the surface at 0 degrees), hydrophilic (partially wetting the surface at 90 degrees), hydrophobic (wetting the surface partially at 90 degrees but not fully at 150 degrees), and superhydrophobic (completely wetting the surface at >150 degrees). Superhydrophobic surfaces (SHSs) is the term used for these substances. Since the lotus leaf, which is mentioned above, is a type of SHS in nature, the droplets simply roll on the surface without any wetting effect.

### 2.2. Young Equation

**Figure 3:**
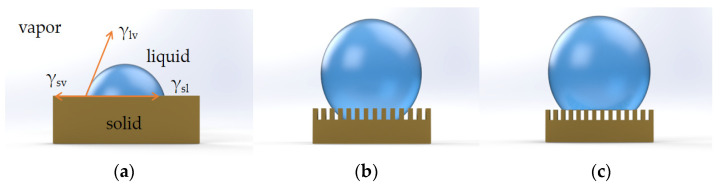
(**a**) Young model. (**b**) Wenzel model. (**c**) Cassie model.

In 1805, Young was the first to propose the concept of contact angle to describe surface wettability, and he was the pioneer of research on wetting [44]. A liquid rests in a droplet on an ideal flat surface, as shown in Figure 3a. The surface free energy of a solid surface is expressed by the Young equation:

(1) $$\gamma_{sv}=\gamma_{sl}+\gamma_{lv}\cos\theta_{Y}$$

where *γ_sv_*, *γ_sl_*, and *γ_lv_* stand for the interfacial energy values for solid–vapor, solid-liquid, and liquid–vapor, respectively, and *θ_Y_* is the contact angle in the Young model. The Young angle is the result of the thermodynamic equilibrium of the surface free energy at the solid–liquid–vapor interface.

### 2.3. Wenzel Model

However, there are not many perfect flat surfaces in the natural world. Wenzel developed the following equation in 1936 to establish a relationship between the macroscopic roughness of a solid surface and the contact angle, explaining how surface roughness increases hydrophobicity [45]:

(2) $$\cos\theta_{W}=r\cos\theta_{Y}$$

where *θ_W_* is the apparent contact angle in the Wenzel model, *r* is the factor of surface roughness, and *θ_Y_* is the contact angle in the Young model. According to Wenzel’s theory, when a liquid comes into contact with a rough surface, it completely fills the voids and grooves of the surface, as seen in Figure 3b; as a result, the static contact angle is decreased, and the sliding angle is increased. Wenzel’s theory states that as roughness increases, hydrophilic surfaces become more wettable, while hydrophobic surfaces become less wettable.

### 2.4. Cassie–Baxter Model

Cassie and Baxter expanded this theory to include rough and porous surfaces in 1944 [46]. It specifies that there are air pockets among the rough grooves and that the rough surface is inherently uneven. As seen in Figure 3c, water droplets adhere to the surface rather than penetrating it. The Cassie–Baxter equation is given by

(3) $$\cos\theta_{CB}=f_{1}\cos\theta_{1}+f_{2}\cos\theta_{2}$$

where *θ_CB_* is the Cassie–Baxter contact angle, *f*_1_ and *f*_2_ are the surface fractions of phases 1 and 2, and *θ*_1_ and *θ*_2_ are the contact angles in phases 1 and 2. As for the air–liquid surface, it can be considered that the air parts of the surface are perfectly non-wetting. So, the water contact angle (WCA) in air is 180°; by taking *θ*_2_ = 180°, Equation (3) can be further reduced to

(4) $$\cos\theta_{CB}=f_{s}\left(\cos\theta_{s}+1\right)-1$$

where *f_s_* is the solid fraction, which means that a fraction of the solid surface is wetted by the liquid.

The relationship between contact angle and surface roughness can be successfully explained by the Wenzel model as well as the Cassie–Baxter model. However, it is important to note that roughness and surface material chemistry should work together on different scales to produce hydrophobicity. Hydrophobic surface preparation, characterization, and application have recently received a lot of attention.

## 3. Magnetron Sputtering Deposition of Hydrophobic Thin Films

There are several types of magnetron sputtering deposition techniques that can be used to deposit thin films, including direct current (DC) magnetron sputtering, radio-frequency (RF) magnetron sputtering, mid-frequency (MF) magnetron sputtering, and high-power impulse (HiPIMS) magnetron sputtering. In DC magnetron sputtering, a constant voltage is applied to the target material, which generates a stable plasma for deposition [47]. RF magnetron sputtering involves the use of a radio-frequency generator to create an oscillating electric field in the chamber, which increases the ionization of the plasma and enhances the deposition rate [18,48]. MF magnetron sputtering, on the other hand, utilizes a moderate-frequency power supply to generate a plasma, providing a balance between the stability of DC magnetron sputtering and the enhanced ionization of RF magnetron sputtering, making it suitable for the deposition of thin films with desired properties [49]. At last, HiPIMS magnetron sputtering uses high-power pulses to create a high-density plasma for thin-film coating, with a higher level of ionization rate of the target [50,51].

Overall, magnetron sputtering deposition is a versatile and widely used technique for the deposition of thin films, with a variety of methods available to tailor the process to specific material and deposition requirements.

There are currently three main types of hydrophobic thin films fabricated using magnetron sputtering: oxides, PTFE, and DLC films. Most studies have focused on improving the hydrophobicity of these films by tuning the magnetron sputtering parameters, or using doping or surface modification. Each material system has unique properties that can meet different application requirements, thus offering a wide range of potential applications.

## 4. Oxide Hydrophobic Thin Films

Oxide materials, such as zinc oxide (ZnO), hafnium oxide (HfO_2_), and cerium oxide (CeO_2_), possess diverse properties, including structural, crystallographic, chemical, morphological, and hydrophobic characteristics, that make them suitable for hydrophobic thin films. Magnetron sputtering is a versatile technique for fabricating hydrophobic oxide thin films, which exhibit excellent chemical stability, thermal stability, and optical properties. The hydrophobicity of oxide thin films can be controlled by adjusting the sputtering process parameters and film microstructure. These hydrophobic oxide thin films have been applied in aerospace, the automotive field, electronic devices, and other fields where their unique properties are desired. Furthermore, their potential for use in high-temperature and high-humidity environments has also been investigated, demonstrating their versatility and potential for challenging conditions.

Several oxides, including cerium oxide, zirconium oxide, zinc oxide, tin oxide, vanadium oxide, hafnium oxide, etc., have been explored for the preparation of hydrophobic surfaces. Table 1 gives a summary of the major results of these reports.

Among these oxides, ZnO thin films are widely used in various applications, such as self-cleaning surfaces, anti-fogging coatings, and corrosion protection. ZnO thin films have aroused great interest because of their outstanding optical properties. A lot of work has been reported so far in order to grow ZnO thin films using different deposition techniques. Magnetron sputtering has emerged as a viable alternative to ion beam sputtering (IBS) for ZnO deposition due to its high deposition rate, industrial applicability, and uniform film formation. The microstructure and properties of ZnO thin films are highly dependent on sputtering parameters such as pressure, power, deposition time, and annealing temperature. By adjusting these parameters, ZnO thin films with various hydrophobic properties can be obtained.

Dave et al. [52] and Malik et al. [53] both focused on the synthesis of ZnO thin films on glass substrates using different sputtering techniques. In the first study by Dave et al., they varied the argon:oxygen gas ratio during deposition and found that decreasing the flow rate of oxygen gas and increasing the flow rate of argon gas resulted in an increase in the average crystallite size, thickness, and surface roughness of the ZnO thin films. They also observed a variation in the WCA of the films. In contrast, Malik et al. varied the sputtering power and working pressure and found that a higher working pressure resulted in an increase in crystallite size and better crystallinity due to an increase in the intensity of the preferred peak (002). They also found that the largest WCA of 120° was observed with a specific combination of sputtering power and pressure, with single-crystal orientation (002) and a maximum average crystallite size of 26 nm. Moreover, modifying the surface chemistry and morphology of ZnO thin films can effectively enhance their hydrophobicity, which can be achieved using techniques such as coating with hydrophobic materials, creating hierarchical surface structures, and plasma treatment.

In addition to research on zinc oxide films, there are also some research teams that have noted the promising hydrophobic properties of thin films made of other metal oxides, including HfO_2_, CeO_2_, TiO_x_, V_2_O_5_, etc.

HfO_2_ exhibits excellent chemical stability and a high dielectric constant, demonstrating exceptional performance in the application of hydrophobic thin films. Dave et al. [54] developed hydrophobic HfO_2_ coatings for outdoor insulators using the DC reactive magnetron sputtering technique. They investigated the effects of sputtering pressure on the properties of nanocrystalline HfO_2_ films, including their geometrical, optical, hydrophobic, and electrical properties. They found that a sputtering pressure of 2.00 Pa produced highly oriented monoclinic HfO_2_ films along the (−111) direction with minimum crystal defects. Moreover, the films deposited at this sputtering pressure exhibited the maximum contact angle (102.3°), band gap (5.32 eV), and film thickness. On the other hand, Jain [55] and co-workers developed HfO_2_ films on a quartz substrate using oblique-angle reactive DC magnetron sputtering. They investigated the effects of the deposition angle on the surface roughness and contact angle of the films. They found that as the deposition angle decreased, the surface roughness increased, and the grain size also increased due to the elongation of grains at low deposition angles. Moreover, they observed that the contact angle increased from 92.3° to 94.6° and then to 106.2° as the deposition angle decreased from 90 degrees to 60 degrees and then to 30 degrees, respectively. Before the angle decreased to 30 degrees, the surface roughness was consistent with the Wenzel model. However, when the angle decreased to 0, the roughness increased, and the contact angle decreased to 94.2° due to the decrease in air pocket formation caused by the larger pore size and the self-shielding effect.

CeO_2_ is a commonly employed hydrophobic thin-film material owing to its unique face-centered cubic lattice structure, which leads to high hardness and chemical stability. CeO_2_ has demonstrated great potential in various applications, for example, functional glass. Zhu [56] and his colleagues successfully fabricated transparent and hydrophobic CeO_2_ films with reliable mechanical properties using magnetron sputtering on glass substrates. The researchers discovered that the WCA of the films was positively related to surface roughness and organic content, while it was negatively related to lattice oxygen (O_I_) content and the O_I_/Ce ratio. As the oxygen flow ratio increased from 14% to 56%, the WCA decreased from 109.1° to 100.4°. After 1-year storage, the researchers conducted a durability test on the contact angle of the cerium oxide films. The results demonstrated that the WCA hardly changed, indicating the excellent durability of the prepared films. Specifically, the contact angle change rates were found to be 0% (104.3° to 103.8°), 14% (109.7° to 109.6°), 28% (107.1° to 106.8°), 42% (102.9° to 101.0°), and 56% (101.9° to 101.3°). Furthermore, the hydrophobic durability of the CeO_2_ films was found to be significantly improved, addressing the previous issue of inadequate durability, and the parameters used for film fabrication were deemed appropriate based on the results obtained. Compared with the previous durability studies, which are rare, its durability is already relatively good.

SnO_2_, also known as tin dioxide, is a semiconducting oxide material that exhibits notable hydrophobicity, or water-repelling properties. This hydrophobicity is due to the presence of low surface energy, which is a result of the material’s unique crystal structure and chemical composition. Much attention has been paid to the combination of SnO_2_ thin films and nanostructures in recent years. Gangwar [57] and colleagues conducted a study on the impact of balanced magnetron (BM) and unbalanced magnetron (UBM) configurations during RF sputtering on the surface properties of SnO_2_ thin films. The study found that the contact angle of SnO_2_ films deposited under the BM configuration increased from 115.9° to 129.6° as the RF power increased from 150 to 250 W. In contrast, the contact angle of SnO_2_ films deposited under the UBM configuration significantly increased from 114.8° to 140.6° with the same increase in RF power, leading to improved surface wettability. The results were attributed to the simultaneous occurrence of various mechanisms under the UBM configuration caused by the bombardment of growing SnO_2_ films with energetic Ar+ ions. Using UBM magnetron sputtering has improved hydrophobicity, which provides great inspiration for method selection during preparation. However, consideration should be given to whether stable and consistent results can be obtained.

With their reversible and controllable surface wettability, V_2_O_5_ thin films have promising applications in areas such as water motion, microfluidic devices, smart membranes, and sensors. Zhang [58] and his group investigated the surface wettability of V_2_O_5_ thin films deposited using RF magnetron sputtering under various conditions. They observed that the wettability of the V_2_O_5_ thin films could be effectively controlled using air storage and heating, and the variation in the WCA could reach up to 73°. It was proposed that the gradual filling of small air molecules into the pores of the film surface could lead to an increase in surface hydrophobicity. After two weeks, the absorption of air molecules reached saturation, and the WCA remained stable. When the samples were reheated, the desorption of air molecules from the film surface occurred, leading to the restoration of superhydrophilicity. Therefore, attention needs to be paid to its long-term hydrophobic stability in applications. Does this also imply hydrophobic instability, or can the tunability of this hydrophobicity be applied in specific fields? Further research and discovery are needed.

Titanium and zirconium oxynitride films are composed of a mixture of oxides, nitrides, and carbides, which gives them unique surface properties that can be tailored to specific applications. Rawal’s group [59] conducted a study on the deposition of TiNO and ZrNO mixture films using the co-sputtering of titanium and zirconium targets with reactive RF magnetron sputtering on Corning1737 glass substrates. The films were found to be sensitive to the power variation of both targets, and it was observed that the structure of the film changed from amorphous oxide to oxynitride films as the power increased. The hydrophobic characteristics of the well-crystalline films led to lower surface-energy values, with the highest WCA reaching 97.5°. The results of this study have important implications for the application of titanium and zirconium oxynitride films in fields such as decorative coatings, anti-corrosion, and sensors. Future research in this field will undoubtedly continue to explore new ways to optimize these materials for specific applications.

Zr_2_ON_2_, which has adjustable properties between ZrN and ZrO_2_, is a ceramic material with a unique combination of properties, including a high refraction index, a wide optical band gap, high hardness, and high chemical stability. Its hydrophobicity has also been explored, making it useful for various applications in areas such as scratch and corrosion resistance. Rawal et al. [60] studied its wettability by conducting a study to investigate the influence of various sputtering parameters on the wettability of Zr_2_ON_2_ thin films deposited using reactive magnetron sputtering. They found that the contact angle and surface roughness of the films increased with an increase in the nitrogen flow rate and deposition time, but an increase in sputtering pressure did not show significant variation in contact angle or surface roughness. These research results reveal the relationship between sputtering parameters and the wettability of Zr_2_ON_2_ thin films, which have potential applications such as self-cleaning surfaces, anti-fouling coatings, and oil–water separation due to their hydrophobic properties.

TiO_x_ is known as a highly biocompatible material, and properly designed hydrophobic thin films can serve two purposes: the mediation of solute adsorption and cell adhesion. Lin’s group [61] conducted a study on the effects of oxygen content on the wettability of titanium oxide films deposited on pure titanium using reactive DC magnetron sputtering. They discovered that increasing oxygen content led to a decrease in WCA due to the formation of hydroxyl radical groups (-OH), which strongly bonded with water molecules. To modify and control surface wettability, the resulting TiO_x_ films were subsequently etched with plasma, leading to a decrease in the WCA by changing the composition of the films from nonstoichiometric to stoichiometric. Moreover, the TiO_x_ film became highly hydrophobic under ultraviolet light but returned to its original relative hydrophobic state under visible light. This effect was attributed to the increase in hydroxyl group contents and dissociative water adsorption on the surface under ultraviolet light and the opposite effect under visible light. This study is very comprehensive and reveals the conclusion that the wettability of TiO_x_ films is influenced by both intrinsic factors of surface chemistry (such as hydroxyl groups) and external factors such as surface roughness. The next step is to optimize the preparation process.

**Figure 4:**
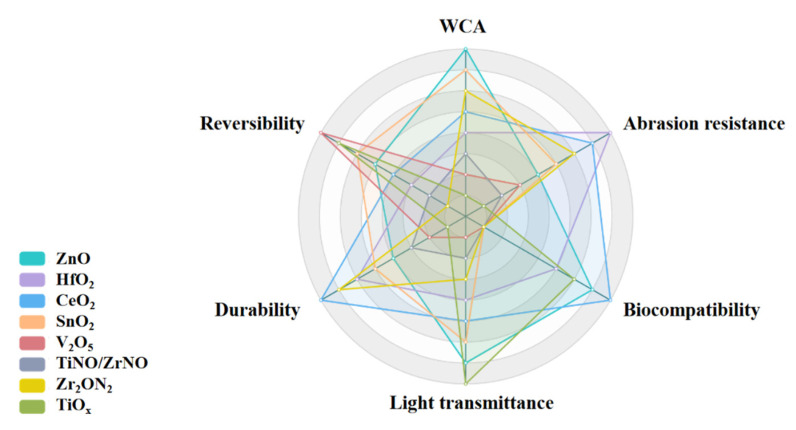
The comprehensive properties of various oxide hydrophobic films prepared using magnetron sputtering.

A radar chart, as shown in Figure 4, shows the comprehensive properties of various oxide hydrophobic films prepared using magnetron sputtering, such as WCA, abrasion resistance, biocompatibility, etc. In the radar chart, the area and shape of the polygon reflect the performance of the film in various performance indicators. The hydrophobicity and various properties of thin films may be influenced by multiple factors, including specific application environments and actual usage conditions. Therefore, the above is only for general reference, and actual situations may vary. In specific applications, it is recommended to conduct practical testing and evaluation to determine the performance of the thin film.

Hydrophobic oxide thin films are highly attractive for a wide range of applications due to their unique properties, such as excellent chemical stability, a high dielectric constant, and tunability. However, achieving desired hydrophobic performance is a challenging task that requires the careful control and optimization of various factors during the preparation process. In order to achieve the widespread application of hydrophobic oxide thin films in diverse fields, it is necessary to conduct further research on their preparation methods, surface chemical composition, and structural characteristics. This would enable us to gain a deeper understanding of the underlying mechanisms governing their hydrophobic behavior and to develop more effective approaches for controlling and optimizing their performance. With continued advancement in this area, hydrophobic oxide thin films hold great promise for a range of applications, from surface protection to electronic devices and beyond. Future research efforts are warranted to elucidate the mechanisms that govern the hydrophobic behavior of oxide films and to further optimize their performance for practical applications.

## 5. PTFE Hydrophobic Thin Films

Efforts to produce fluorocarbon polymer-like films began in the late 1960s and continue to this day. Polytetrafluoroethylene (PTFE) possesses several desirable properties, including high thermal stability, low dynamic friction coefficient, chemical inertness, a low dielectric constant, and high hydrophobicity, making it an attractive material for various technical applications. Its widespread commercial use makes it an excellent candidate for developing hydrophobic layers, anti-reflective and protective coatings, lubricants, chemical containers, and chemically resistant molded parts. Many researchers have prepared different hydrophobic films from PTFE materials. A summary of the main results of these reports is given in Table 2.

PTFE films have the lowest refractive index among known polymers, which directly indicates the possibility of using this material for fiber optic applications and as a top layer for existing anti-reflective coatings. Tripathi et al. [62] studied the properties of ultrathin PTFE films prepared using magnetron sputtering on BK7 glass substrates. ATR-FTIR measurements showed that the films consisted of C-C and C-F_x_ (x = 1, 2, 3) bonds, with an F/C ratio of ~1.5, indicating hydrophobic properties. CF_3_ and CF_2_ radicals led to a reduction in the surface free energy of the films, resulting in hydrophobicity. Meanwhile, in order to improve the adhesion on the glass substrate, Kim [23] coated PTFE film using the catalytic chemical vapor deposition (Cat-CVD) method on RF-sputtered PTFE film and then obtained a superhydrophobic surface with a 153° ± 1 WCA and good adhesion on the glass substrate. As mentioned above, it can be seen that PTFE films can combine excellent hydrophobic performance and optical properties and exhibit good adhesion to glass.

Polyethylene terephthalate (PET) is a common plastic substrate material with good optical transparency, chemical stability, and mechanical strength. It is widely used in various fields, such as packaging, optical films, and electronic devices. When applying PTFE film to a PET substrate, the PET substrate usually does not have a significant, negative impact on the hydrophobicity of the PTFE film. Satulu et al. [63] prepared a hydrophilic/hydrophobic composite membrane by depositing PTFE films onto one side of a PET track-etched membrane using RF magnetron sputtering. The WCA increased by 20° after the deposition of the PTFE films due to the diffusion of fluorine-containing radicals along the pore channels of the membrane. Becker et al. [64] combined magnetron sputtering with a Nd:YAG laser to deposit PTFE thin films onto PET substrates and obtain additional properties. Under the optimized conditions, a maximum static contact angle of 160.8° ± 2.7 was finally obtained. By combining two preparation methods, the hydrophobicity was greatly improved, which can broaden the application field of PTFE film. However, the cost of preparation needs to be considered, which may become a difficult problem for industrialization.

Kylián et al. [65] conducted a time-efficient process in which a superhydrophobic coating was deposited using C:H nanoparticles, taking only 2 min, followed by a PTFE-like overlay in just 1 min. They used a two-step process for producing surfaces with well-defined roughness, which involved the deposition of nanoparticle films using gas aggregation sources, followed by coating the pre-prepared films with RF magnetron-sputtered fluorocarbon films. They found that a roughness greater than 60 nm was required to achieve superhydrophobic and slippery surfaces, with values of WCA close to 180° being observed for the roughest samples (RMS > 133 nm). The highlight of this research is its extremely high efficiency and a contact angle of up to 180°, which can be considered to indicate excellent film. It should be noted that the actual hydrophobic state is not only determined by RMS roughness but also strongly depends on surface chemical properties and surface nanostructures, such as the shape, size, density, and spacing of nanostructures.

In addition to PTFE films exhibiting hydrophobic properties on glass and PET substrates, researchers have prepared such films on silk with magnetron sputtering. In the study by Huang’s group [66], a magnetron-sputtered PTFE coating was applied to improve the hydrophobic property of silk fabric. The contact angle of the PTFE-coated fabric showed a significant increase from 68° to about 138°. The higher argon pressure increased the number of collisions, causing higher ionization efficiency and plasma density, leading to an increase in roughness. The study indicated that the working pressure had little effect on the static contact angle of the fabric due to the formation of stable chemical structures on the fabric surface after PTFE sputter-coating.

As a high-quality semiconductor material, silicon wafers have very high flatness and adaptability. This is critical for the properties of the film, which were affected by the properties of the substrate. The flatness of the silicon wafer contributes to a uniform, tight, and dense PTFE film. The preparation of PTFE films on silicon wafers can be adapted to existing semiconductor processes and facilitate integration into a variety of microelectronic and optoelectronic devices, such as optical coatings, microfluidic chips, etc.

Kylián et al. [67] attempted to deposit thin, superhydrophobic PTFE films by means of the RF magnetron sputtering of a PTFE target. They found that the F/C ratio of the deposited films could be obtained under high pressures or at longer distances from the sputtered target. The density of CF2 was drastically reduced as the distance from the target increased, allowing the free radicals to have more time to diffuse on the surface and leading to the formation of nuclei and surface roughness.

Improving the durability of hydrophobic PTFE films and extending their service lives is a problem that needs to be solved. The durability of hydrophobicity was not commonly covered in previous review articles. In Drabik’s [68] study, PTFE films were prepared onto silicon wafers using RF magnetron sputtering. These films possessed static contact angles of water ranging from 112° to ~170°. It has been observed that SHF films with stable structures remained superhydrophobic even after 12 months when stored under standard room conditions (temperature, relative humidity, pressure, etc.). The PTFE film in this study can maintain excellent performance for a long time even in complex practical application environments, which may provide good enlightenment relatively to the hydrophobic durability problem in industrialization.

Yu et al. [69] conducted a comprehensive study on the atomic bonding, growth mode, and surface morphology of PTFE thin films on silicon wafers prepared using RF magnetron sputtering at different nominal deposition powers and thicknesses. The WCAs of these PTFE thin films were measured and found to range from 107° to 116°, which are higher than the ~100° angle reported by Tripathi et al. for PTFE films deposited on transparent glass. Moreover, the WCAs measured on fluorocarbon plasma polymer films approached 105° in their study.

In a work by Jafari et al. [70], a superhydrophobic surface with low contact angle hysteresis was created by sequentially anodizing aluminum and sputtering PTFE. The RF-sputtered PTFE coating demonstrated the very low contact angle hysteresis of 3° and a high static contact angle of 165°. High amounts of CF_3_ and CF_2_ groups, which were found with X-ray photoelectron spectroscopy (XPS) studies, were what gave the coatings their hydrophobic nature. The performance of this superhydrophobic film was examined under atmospheric icing conditions. These results showed that the strength of ice adhesion on superhydrophobic surfaces was 3.5 times lower than on the polished aluminum substrate.

In order to meet the functions of anti-frosting, anti-icing, transparency, and other functions of smart windows for buildings and automobiles, Lee et al. [71] demonstrated the fabrication of multifunctional electrodes, including a Ag network with thin PTFE films for flexible transparent thin-film heaters (TTFHs), using MF magnetron sputtering. Such a properly designed surface should exhibit self-cleaning, waterproof, highly transparent, and flexible properties. With a PTFE thickness of 10 nm, the Ag network passivated with the hydrophobic PTFE layer showed a high contact angle of 102.42°. Compared with the bare Ag network with a contact angle of 65.05°, the Ag network and PTFE films exhibited significantly increased contact angles greater than 100°, regardless of the PTFE thickness. Meanwhile, Lee et al. [72] prepared superhydrophobic Ag nanowire (NW) network electrodes passivated with PTFE using MF magnetron sputtering to realize self-cleaning and TTFHs. Compared with bare Ag-NW film without a PTFE layer, which had a WCA of 46°, the addition of 75 nm thick PTFE film resulted in a significant improvement, with the contact angle reaching a maximum value of 106°. This improvement is beneficial for transparent TFH-based smart windows.

However, PTFE lacks mechanical stability and adhesion to many substrates, which limits its practical application. To address this issue, researchers have explored various strategies to improve the mechanical properties and adhesion of PTFE films. When designing a film structure, we can consider using a multilayer structure or adding an intermediate layer to improve the adhesion of the film to the substrate or other materials. Liao et al. [73] fabricated ZnO/SiO_2_/PTFE sandwich-nanostructure superhydrophobic thin films, which were prepared on glass using the RF magnetron sputtering technique. These authors showed that a contact angle of up to 167.2° was obtained, attributing this to the low surface energy of PTFE and the micro and nano air pockets induced by the rough nanostructures.

**Figure 5:**
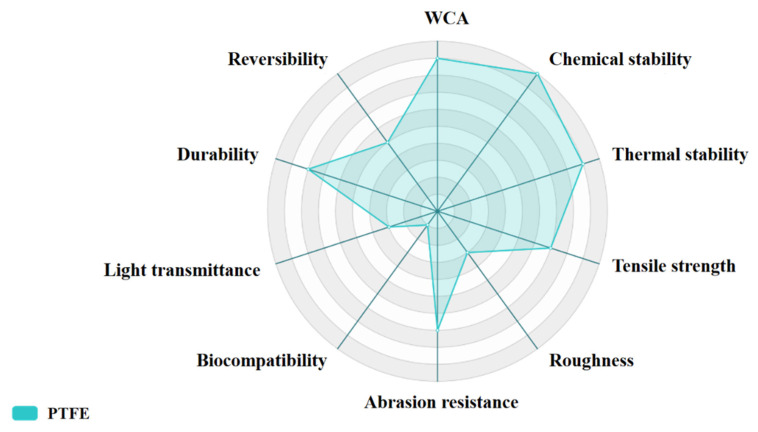
The comprehensive properties of PTFE hydrophobic films prepared using magnetron sputtering.

The radar chart in Figure 5 shows the comprehensive properties of PTFE hydrophobic films prepared using magnetron sputtering, such as chemical stability, thermal stability, etc. The advantages and disadvantages of PTFE in various dimensions can be clearly seen, which is a good inspiration for thinking about how to combine and improve the performance of PTFE film. Researchers can improve performance by combining magnetron sputtering with other methods, changing the substrate, or preparing composite thin films that can be applied in various application fields. However, it should be emphasized that the information on the chart is for reference only, and the actual performance index is affected by multiple factors and may vary.

In summary, magnetron sputtering is a promising technique for preparing PTFE coatings with excellent hydrophobic properties. The properties of the coatings can be tailored by adjusting the deposition conditions, such as pressure, distance from the target, and deposition time. To address the limitations of PTFE coatings, researchers have explored various strategies to improve the mechanical properties, adhesion, and self-healing ability of PTFE coatings. These advancements are expected to expand the practical applications of PTFE coatings in various fields.

## 6. Diamond-like Carbon Hydrophobic Thin Films

Diamond-like carbon (DLC) films have been studied for decades as effective protective coatings, because of their excellent properties such as high hardness, low friction coefficient, high wear resistance, chemical stability, corrosion resistance, thermal conductivity, electrical insulation, optical permeability, and biocompatibility. The film is mainly amorphous carbon consisting of a network of sp^3^- and sp^2^-bonded carbon atoms. DLC films can be widely used in mechanical, electronic, optical, chemical, and biological fields. DLC films have attracted interest as protective coatings for medical implants such as hip joints, knee joints, and coronary stents, and as replacement in mechanical devices. 

However, pure DLC films have many drawbacks, such as high internal stress, low oxidation resistance, poor adhesion force, and hydrophilic surface. From this point of view, doped DLC films have recently attracted much attention. It is well known that all of these properties can be changed within a specific range with the addition of other elements to DLC. Incorporating other elements, such as Ti, Si, W, Cr, etc., into DLC films provides an effective way to improve the properties of the films.

Unfortunately, in the beginning, much of research focused on improving mechanical properties. However, more and more researchers are noticing the influence of element doping in DLC films on other properties, especially hydrophobicity, because in the biomedical field, the hydrophobicity of DLC films strongly influences the biological behavior of the implant. The researchers made different kinds of doped-DLC hydrophobic films, such as doped with silver, aluminum, niobium, etc., and their results are summarized in Table 3.

Several different research groups have investigated the changes in surface properties of DLC films after adding a third element into them, such as Al, Ni, and Ag. Choi’s group [74] studied the characteristic of Ag-doped DLC films prepared using a silver DC magnetron sputter source. As the concentration of Ag in DLC films increased, the contact angle increased from 80 to 99° in case of distilled water and from 61 to 95° in case of formamide, respectively. The reductions in the total surface energy of Ag-incorporated DLC films were attributed to the reduction in polar and dispersive components. It could be seen from Raman spectroscopy that the increase in Ag contents in DLC films resulted in more sp^2^ bonding in the amorphous carbon matrix.

Ding et al. [75] performed a similar study in 2022, investigating the influence of bias voltage effects on the properties of Al-doped DLC films prepared with a hybrid deposition technique combining high-power impulse magnetron sputtering and pulsed direct current magnetron sputtering. The results revealed that the maximum value of the WCA was up to 120.9° at −300 V bias voltage. It then slightly decreased to 117.8° with an increase in the bias voltage to −400 V, but all films revealed hydrophobic performance. It indicated that excellent hydrophobicity could be obtained by adjusting the bias voltage in DLC films.

In 2021, Ding et al. [76] studied the effect of bias voltage on the properties of DLC films with Nb doping and confirmed that bias voltage had an evident impact on the surface wettability of films, the degree of disorder of the carbon structure in films was intensified, and the sp^3^-C bond fraction was improved with an increase in bias voltage. They explained it in several aspects. First, the surface roughness decreased with the increase in bias voltage; thus, the corresponding contact angle decreased. Second, the sp^2^-C-rich surface could decrease the dangling bonds in order to lower the surface energy of films. Consequently, the hydrophobicity improved. At last, the increase in metal content in films caused by the bias voltage could also minimize the WCA.

In addition to silver, researchers have also used titanium to dope. DLC film has high hardness, but due to its amorphous structure and low thickness, it is relatively susceptible to mechanical damage, such as scratches, wear, etc., which affects its durability and service life. Ma et al. [77] prepared Ti-doped DLC films on Ti alloys using reactive magnetron sputtering combined with PSII technology. The result showed that the coarse surface morphology produced with ion beam bombardment and the low surface energy due to Ti element doping improved the hydrophobicity of DLC films. Owing to Ar and Ti ion bombardment and Ti and C ionized-cluster deposition in this experiment, the WCA could reach 104.2 ± 1°, and the mechanical properties of Ti-doped DLC film were also significantly improved.

Lubwama et al. [78] studied the hydrophobic characteristics of DLC and Si-DLC films with and without Si-C interlayers, which were deposited on nitrile rubber using a closed-field unbalanced magnetron sputtering ion plating system. The WCA measured for all the coatings varied between 101° and 105°, indicating the hydrophobic properties of the films deposited on the nitrile rubber substrate. Hydrophobicity resulted from sp2 and sp3 hybridized carbon bonds in the DLC films. A higher surface free energy was observed for Si-DLC with the Si-C interlayer (with lower WCA) than for DLC with the Si-C interlayer (with higher WCA).

Pfleging et al. [79] deposited amorphous carbon layers doped with hydrogen on silicon wafers using reactive direct current magnetron sputtering. Films with three different hydrogen contents were synthesized. After deposition, they utilized UV laser material processing at the wavelength of 193 nm or 248 nm to perform chemical surface modification and surface structuring on micro- and nanometer scales. Unprecedented changes in surface energy and WCA were found with the increase in hydrogen concentration in the films. The contact angle of films with 48 at% hydrogen was reduced from 95° to 15°. Direct laser structuring at high laser fluences revealed the improvement in wettability induced by micro- and nano-sized structures. Superhydrophobic behavior (WCA = 160°) was attained with patterns using square structures with a width of 150 μm. 

Lee’s group [80] found a highly hydrophobic surface feature in hydrogenated Cu-incorporated diamond-like carbon (a-C:H/Cu) films prepared with a radio-frequency plasma magnetron sputtering system at various CH_4_/Ar gas ratios. According to the contact angle goniometer, the contact angle of the a-C:H film was 80 ± 2.2°. The a-C:H/Cu-3 sample possessed the highest contact angle of 115 ± 2.5°. Although the a-C:H/Cu-1 sample presented a lower contact angle (104 ± 3.2°) than that of the a-C:H/Cu-2 and a-C:H/Cu-3 samples, it still classifies as a hydrophobic surface because the angle exceeds 90°. By nature, copper embedded in a-C:H films is a highly hydrophobic surface feature.

Tsai et al. [81] deposited Cu/a-C:H films on SUS 304 stainless-steel substrates using a hybrid deposition process that combines RF magnetron sputtering and plasma-enhanced chemical vapor deposition in different atmospheres comprising different proportions of Ar/CH_4_ mixture. It was shown that the films obtained in the 14.7% CH_4_ atmosphere were rougher than those obtained in the 45.4% CH_4_ atmosphere, which indicates that the highest Cu content in the films was obtained in the 14.7% CH_4_ atmosphere, and it could be one of the reasons for the better hydrophobic properties of the films. As shown in the figure, the maximum WCA could reach more than 130° when in the 14.7% CH_4_ atmosphere.

**Figure 6:**
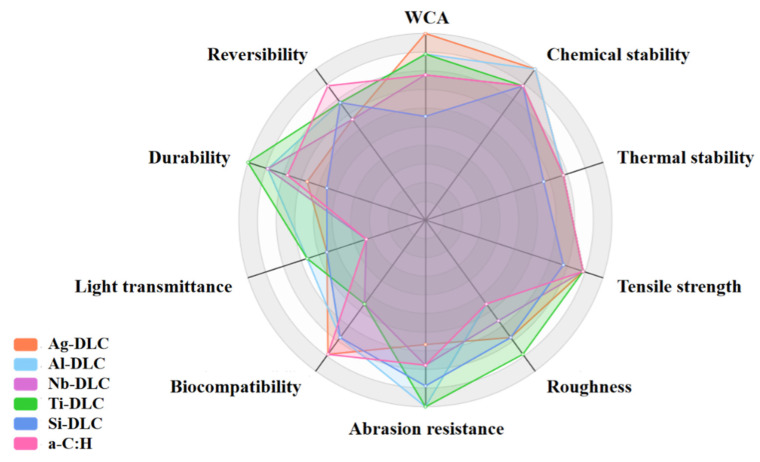
The comprehensive properties of various DLC hydrophobic films prepared using magnetron sputtering.

The radar chart shown in Figure 6 represents the comprehensive properties of various DLC hydrophobic films doped with different elements, such as light transmittance, durability, etc. These properties all have some relationship with hydrophobicity. The evaluation of this figure is for reference only, and film performance is affected under different preparation and use conditions.

Compared with some traditional coating materials, DLC films are expensive to prepare due to the requirements of highly controlled equipment and process conditions, including high-vacuum systems, high-energy sources, complex deposition processes, and equipment maintenance. However, DLC films are widely used in specific application fields, such as automotive parts, aerospace, and medical devices, due to their unique performance and characteristics, which can provide significant added value and performance improvement. Therefore, in these high-value-added applications, the preparation cost of DLC films may be acceptable. When considering the use of DLC films in specific applications, it is essential to carefully weigh their advantages and disadvantages and consider additional performance improvements. Appropriate materials and process conditions should be selected based on the needs and application requirements.

## 7. Other Promising Materials

There has been significant advances made in the field of bio-ceramics, particularly hydroxyapatite (HA), during the past 20 years. Early in 2011, in Thian et al.’s work [82], silicon-substituted hydroxyapatite (SiHA) thin coatings of varying Si compositions were deposited on Ti substrates using a magnetron co-sputtering technique. This interdisciplinary paper demonstrated that increased bioactivity was related to surface wettability. These thin films have improved mechanical and biological properties, including increased cell adhesion and proliferation, making them suitable for use as implant coatings. Because radio-frequency magnetron sputtering has numerous advantages, such as high efficiency, favorable bonding strength, and controllable properties, an increasing trend has emerged for utilizing its benefits in the fabrication of HA bio-coatings [83]. Depending on current requirements and objectives, it is possible to tune the characteristics of these layers in a way that facilitates successful biomedical applications.

Sputtering techniques have been used to create multilayer coatings such as polymer/metal/polymer or polymer/metal/transparent conducting oxide (TCO) films, in addition to single-layer materials with hydrophobic properties. In Kang et al.’s work [84], the polymer/metal/polymer and polymer/metal/inorganic transparent conducting thin films produced using a continuous roll-to-roll sputtering process on a large-area flexible polymer substrate exhibited excellent electrical properties and visible-light transmittance. They also had water-repellent surfaces to help prevent wetting and contamination. These coatings exhibited unique properties, such as high transparency, conductivity, and hydrophobicity, which make them suitable for applications in solar cells, electronic devices, and self-cleaning surfaces. The use of multilayer coatings is a promising area of research with potential applications in various industries, including energy, electronics, and manufacturing.

The two materials described above have great potential for future development. Silicon-substituted hydroxyapatite thin films have been widely studied and applied in various fields. Multilayer hydrophobic thin films, on the other hand, are a relatively novel material with unique properties such as high transparency, high conductivity, and superhydrophobicity, making them suitable for applications in solar cells, electronic devices, and self-cleaning surfaces, among others. Although some progress has been made in the preparation of hydrophobic thin films using magnetron sputtering, there are still many challenges to be overcome. More efforts are needed to improve the performance and stability of these materials in order to promote their practical applications. We are confident in the prospects and development of these materials and believe that they will bring more opportunities and achievements for future technological and industrial development.

## 8. Summary and Outlook

Hydrophobic thin films prepared using magnetron sputtering deposition have gained significant attention due to their potential applications in various fields, such as anti-fouling coatings, self-cleaning surfaces, and biomedical devices. Despite the considerable advancements in hydrophobic thin films, several gaps in comprehending the fundamental mechanisms underlying their properties still need to be addressed. Specifically, the trade-off among film structure, surface chemistry, and hydrophobicity needs to be explored and developed. Moreover, further studies are necessary to systematically investigate the effects of deposition parameters, including substrate temperature and pressure, on film properties. Therefore, more comprehensive investigations are warranted to bridge the existing knowledge gaps and facilitate the development of highly efficient hydrophobic thin films.

There is still a need to explore and develop the technology further. It is essential to propose potential future directions for research in this area: Firstly, scientists could concentrate on developing more durable and stable hydrophobic thin films. Secondly, investigating the surface properties of hydrophobic coatings could provide insights into how they interact with water and other liquids. Advanced surface characterization techniques could be utilized to study these coatings’ roughness, chemical composition, and topography. Thirdly, new applications for hydrophobic films could be explored. While these coatings have already been found to be used in many applications, researchers can investigate their new potential in other fields, such as energy harvesting and microfluidics. Fourthly, researchers could focus on developing eco-friendly films due to current hydrophobic coatings relying on toxic or environmentally harmful materials. Lastly, hydrophobic thin films could be combined with other functional coatings to create multifunctional surfaces with various valuable performance. For instance, combining hydrophobic coatings with anti-corrosion or anti-reflective coatings could endow surfaces with multiple functionalities.

In conclusion, the potential directions for research on hydrophobic thin films prepared using magnetron sputtering deposition are vast, and continued innovation in this field could lead to new and exciting advances for these coatings. We believe that continued innovation in this field can lead to new and exciting applications for these thin films.

## Figures and Tables

**Table 1:** Summary of references on oxide thin films.

Material	Method	Preparation Condition	Hydrophobic Properties	Substrate	Reference
ZnO	RF magnetron sputtering	1. RF power: 150 W 2. Working pressure: 1.0 Pa 3. Working gases: Ar/O_2_	WCA: 98.3~102.1°	Corning glass	Dave [52]
ZnO	DC magnetron sputtering	1. Working pressure: 2.67~18.66 Pa 2. Sputtering time: 20 min 3. Sputtering power: 40–80 W 4. Working gases: Ar:O_2_: 3:4 (sccm)	WCA maximum of 120° at 10.67 Pa working pressure	Glass	Malik [53]
HfO_2_	DC reactive magnetron sputtering	1. Working gas: Ar 2. Reactive gas: O_2_ 3. Working pressure: 0.67~3.33 Pa 4. Sputtering power: 50 W	WCA maximum of 102.3° at 2.00 Pa	Glass and quartz	Dave [54]
HfO_2_	Oblique-angle reactive DC magnetron sputtering	1. Working gases: Ar/O_2_ 2. Sputtering power: 100 W 3. Sputtering pressure: 1.33 Pa	WCA of 106.3° at deposition angle of 30° is the highest so far for HfO_2_ thin films	Quartz	Jain [55]
CeO_2_	Magnetron sputtering	1. Working pressure: 0.34 Pa 2. Working gas: Ar 3. Reactive gas: O_2_ 4. Bias voltage: −60 V	WCA maximum of 109.1° at an oxygen flow ratio of 14%	Glass	Zhu [56]
SnO_2_	Customized downstream RF magnetron sputtering system using balanced and unbalanced magnetron configurations	1. Sputtering power: 150~250 W 2. Working pressure: 4.00 Pa 3. Deposition time: 60 min 4. Working gases: Ar:O_2_ = 8:2	At RF power of 250 W, maximum of contact angle value of 140.6°	Si (100)	Gangwar [57]
V_2_O_5_	RF magnetron sputtering	1. Working gases: Ar/O_2_ 2. Working pressure: 1.33 Pa 3. Deposition time: 3 h~17 h 4. Sputtering power: 200 W	Reversible and controllable by storing in air, or heating, with a maximum WCA difference of 73°	Si (100)	Zhang [58]
TiNO and ZrNO	Reactive RF magnetron sputtering	1. Sputtering pressure: 2 Pa 2. Working gas: He 3. Reactive gases: O_2_ and N_2_	WCA maximum: 97.5°	Corning 1737 glass	Rawal [59]
Zr_2_ON_2_	Reactive magnetron sputtering	1. Working gases: N_2_/O_2_/He 2. Sputtering power: 150 W 3. Working pressure: 2 Pa	WCA maximum of 104° after 140 min sputtering time	Glass	Rawal [60]
TiO_x_	Reactive DC magnetron sputtering	1. Working gases: Ar/O_2_ 2. DC currents: 0.5 A/1.0 A	WCA can freely change in 0°~90°	Commercially pure titanium disks (99.99%)	Lin [61]

**Table 2:** Summary of references on PTFE thin films.

Material	Method	Preparation Condition	Hydrophobic Properties	Substrate	Reference
PTFE	RF magnetron sputtering	1. Sputtering power: 160 W 2. Working gas: Ar 3. Working pressure: 1.8 × 10^−1^ Pa	Contact angle was nearly equal to 100°	BK7 Glass	Tripathi [62]
PTFE	RF magnetron sputtering and Cat-CVD methods	1. Sputtering power: 50 W 2. Working gas: Ar 3. Working pressure: 2.67 × 10^−1^ Pa	WCA is 122.3°, with good adhesion on the glass substrate	Glass slides	Kim [23]
PTFE	RF magnetron sputtering	1. Working gas: Ar (100 sccm) 2. Sputtering power: 50 W 3. Working pressure: 6.8 × 10^−1^ Pa	Contact angle on PET was 20° more than that on Si wafer (105 ± 20°)	PET track-etched membrane	Satulu [63]
PTFE	Innovative laser-assisted magnetron sputtering	Not mentioned	Maximum static contact angle value: 160.8 ± 2.7°	PET	Becker [64]
PTFE	RF magnetron sputtering	1. Working gas: Ar (5–50 Pa, 7 sccm) 2. Sputtering power: 200 W	173° at 25 cm distance	Not mentioned	Kylián [65]
PTFE	RF magnetron sputtering	1. Working gas: Ar (as the bombardment gas) 2. Working pressure: 5~50 Pa	Maximum of static contact angle of 139° at 50 Pa	Silk twill fabric with a mass of 36 m/m (145 g/m^2^)	Huang [66]
PTFE	RF magnetron sputtering	1. Working gas: Ar 2. Working pressure: 5 Pa 3. Sputtering power: 100 W	Maximum static contact angle value: close to 180°	Si wafers	Kylián [67]
PTFE	RF magnetron sputtering	1. Working gas: Ar 2. Working pressure: 5~85 Pa 3. Sputtering power: 200 W 4. Distance between target and substrate: 14 cm/25 cm	WCA value at 50 Pa and 25 cm distance after 20 min above 170° (superhydrophobic)	Polished silicon substrate	Drabik [68]
PTFE	RF magnetron sputtering	1. Working gas: Ar 2. Sputtering power: from 120 to 200 W 3. Working pressure: 1 Pa	WCA ranged from 107° to 116°	Silicon wafer	Yu [69]
PTFE	RF magnetron sputtering	1. Working gas: Ar 2. Sputtering power: 50 W 3. Working pressure: 2.66 Pa	WCA was up to 165°	An anodized aluminum surface	Jafari [70]
PTFE	MF magnetron sputtering system	1. Sputtering power: 150 W 2. Working gas: Ar 3. Ar flow rate: 50 sccm 4. Working pressure: 1.20 Pa	Regardless of the PTFE thickness, contact angle was greater than 100°	Ag network	Lee [71]
carbon nanotubes and a PTFE	Mid-frequency magnetron sputtering	1. Sputtering power: 300 W 2. Working gas: Ar 3. Ar flow rate: 50 sccm 4. Working pressure: 0.93 Pa	At a PTFE thickness of 75 nm, the PTFE/Ag NW electrode demonstrated the highest contact angle of 106°	Ag NW/PET and Ag NW/PU	Lee [72]
ZnO/SiO_2_/PTFE sandwich-nanostructure	RF magnetron sputtering	1. Working gas: Ar 2. Working pressure: 1.5 Pa 3. Sputtering power: 100 W	Contact angle was up to 167.2°	Glass slides	Liao [73]

**Table 3:** Summary of references on DLC thin films.

Material	Method	Preparation Condition	Hydrophobic Properties	Substrate	Reference
Ag-DLC	A hybrid deposition system composed of an end-hall-type hydrocarbon ion gun and a silver DC magnetron sputter source	1. Working gas: Ar 2. Sputtering power: 298 W 3. Working pressure: 0.08~0.15 Pa 4. DLC sputtering bias voltage: −800 V 5. DLC sputtering time: 8 min 6. Ag-DLC sputtering bias voltage: −200 V 7. Ag-DLC sputtering time: −200 V	WCA: 80~99 Formamide contact angle: 61~95°	P-type Si (100) wafer	Choi [74]
Al-DLC	HiPIMS and PDC	1. Working gas: Ar 2. Working pressure: 0.5 Pa 3. Bias voltage: 0~−400 V	WCA was up to 120.9° when bias voltage was −300 V	Silicon	Ding [75]
Nb-DLC	High-power impulse magnetron sputtering (HiPIMS) unit and pulse direct current magnetron sputtering source (PDC)	1. Working pressure: 0.55 Pa 2. Bias voltage: 0, −50, −100, −200, and −300 V 3. Working gas: Ar	WCA was up to 103° when bias voltage was −50 V	Well-polished SUS 304 and (100) Si wafers	Ding [76]
Ti-DLC	Reactive magnetron sputtering combined with PSII technology	1. Working pressure: 0.07 Pa 2. Substrate bias voltage: −100 V 3. Working gas: Ar	WCA was up to 104.2 ± 1°	Ti_6_Al_4_V	Ma [77]
Si-DLC	Closed-field unbalanced magnetron sputtering ion plating (CFUBMSIP) system	1. Substrate voltage bias: −30 V 2. Working gases: Ar/C_4_H_10_ = 12 sccm:8 sccm 3. Working current: 0.5 A	WCA: 101~105°	Etched acrylonitrile butadience rubber (NBR)	Lubwama [78]
Amorphous hydrogen-doped DLC (a-C:H)	Reactive DC magnetron sputtering and laser-assisted chemical modification	1. Working gases: Ar/CH_4_ 2. Working pressure: 0.6 Pa	WCA was up to 160° at low c(CH4) = 5.7%	Silicon wafers	Pfleging [79]
Hydrogenated Cu-incorporated DLC (a-C:H/Cu)	RF magnetron sputtering	1. Working pressure: 1.08 Pa 2. Working gases: CH_4_/Ar	WCA was up to 115 ± 2.5°	Glass	Lee [80]
Cu/a-C:H films	RF magnetron sputtering and plasma-enhanced chemical vapor deposition	1. Working gases: methane–argon gas mixture 2. Working pressure: 226.7 Pa	WCA was up to 130° at 14.7% CH_4_	SUS 304 stainless-steel substrate	Tsai [81]

## Data Availability

Not applicable.
